# Needs in Nursing Homes and Their Relation with Cognitive and Functional Decline, Behavioral and Psychological Symptoms

**DOI:** 10.3389/fnagi.2016.00072

**Published:** 2016-04-21

**Authors:** Ana Rita Ferreira, Cláudia Camila Dias, Lia Fernandes

**Affiliations:** ^1^Department of Health Information and Decision Sciences (CIDES), PhD Program in Clinical and Health Services Research (PDICSS), Faculty of Medicine, University of PortoPorto, Portugal; ^2^Department of Health Information and Decision Sciences (CIDES), Center for Health Technology and Services Research (CINTESIS), Faculty of Medicine, University of PortoPorto, Portugal; ^3^Department of Clinical Neurosciences and Mental Health, Faculty of Medicine, Center for Health Technology and Services Research (CINTESIS), University of Porto, Clinic of Psychiatry and Mental Health, CHSJPorto, Portugal

**Keywords:** needs assessment, CANE, elderly, nursing home, dementia, depression, behavioral and psychological symptoms, functional dependency

## Abstract

Unmet needs are becoming acknowledged as better predictors of the worst prognostic outcomes than common measures of functional or cognitive decline. Their accurate assessment is a pivotal component of effective care delivery, particularly in institutionalized care where little is known about the needs of its residents, many of whom suffer from dementia and show complex needs. The aims of this study were to describe the needs of an institutionalized sample and to analyze its relationship with demographic and clinical characteristics. A cross-sectional study was conducted with a sample from three nursing homes. All residents were assessed with a comprehensive protocol that included Mini-Mental State Examination (MMSE), Geriatric Depression Scale (GDS-15), Neuropsychiatric Inventory (NPI) and Adults and Older Adults Functional Inventory (IAFAI). To identify needs, the Camberwell Assessment of Need for the Elderly (CANE) was used. The final sample included 175 residents with a mean age of 81 standard deviation (SD = 10) years. From these, 58.7% presented cognitive deficit (MMSE) and 45.2% depressive symptoms (GDS). Statistically significant negative correlations were found between MMSE score and met (*r*_s_ = −0.425), unmet (*r*_s_ = −0.369) and global needs (*r*_s_ = −0.565). Data also showed significant correlations between depressive symptoms and unmet (*r*_s_ = 0.683) and global needs (*r*_s_ = 0.407), and between behavioral and psychological symptoms (BPSD) and unmet (*r*_s_ = 0.181) and global needs (*r*_s_ = 0.254). Finally, significant correlations between functional impairment and met (*r*_s_ = 0.642), unmet (*r*_s_ = 0.505) and global needs (*r*_s_ = 0.796) were also found. These results suggest that in this sample, more unmet needs are associated with the worst outcomes measured. This is consistent with previous findings and seems to demonstrate that the needs of those institutionalized elderly remain under-diagnosed and untreated.

## Introduction

With an aged population and the resultant increase of chronic diseases, including dementia, in the near future, the evaluation of the emergent needs of this population has become crucial (Cadieux et al., 2013). In fact, people are not only living longer, but also presenting important age-related diseases which are often chronic and associated with daily functional and mental limitations (Fahy and Livingston, 2001), which can eventually lead to their institutionalization (van der Ploeg et al., 2013). Therefore, institutionalization rates increase when elderly dependency levels and needs become too complex or costly to be met at home (Hancock et al., 2006; Orrell et al., 2007) or by the available community services. As a result, people living in long-term care settings often present dementia and other concomitant comorbid diseases that will not only increase their morbidity and mortality, but also result in multiple and complex needs in these care settings (Martin et al., 2002; Hancock et al., 2006; Orrell et al., 2007; Cadieux et al., 2013).

In this context, higher demands are imposed on nursing homes and other long term care facilities (Alzheimer’s Association, 2014) as their residents are becoming older, frailer and more dependent (Stern et al., 1993; Martin et al., 2002) due to their physical and cognitive impairments (van der Ploeg et al., 2013). The way their complex needs are met is relatively unknown (Hancock et al., 2006), particularly if one considers older people with dementia (Lee et al., 2002) that often have their needs unmet (Cohen-Mansfield and Mintzer, 2005; Orrell et al., 2008), despite the importance of planning their care. In fact, there is scarce evidence on how far these institutions can identify and address the needs of their residents. However, it is well known that some of those needs may be neglected for a number of reasons, including their major complexity, the progressive inability of patients to express themselves (Mozley et al., 1999), or even due to resignation and hopelessness, leaving them as unmet needs (Holmquist et al., 2003; Hancock et al., 2006).

An unmet need is described as a problem for which an individual is not receiving an appropriate assessment or intervention that could potentially meet the need (Iliffe et al., 2004; Orrell and Hancock, 2004). Needs, particularly when unmet, are found to be important clinical and research targets. They lead to a decrease in quality of life (Slade et al., 2005; Hoe et al., 2006), higher anxiety, depression and challenging behaviors (Hancock et al., 2006), as well as predicting premature institutionalization and mortality (Gaugler et al., 2005). Once patients are in long-term care settings, unmet needs have been found to be associated with increased distress (Hoe et al., 2006; Orrell et al., 2008) and dissatisfaction with services. Research has also demonstrated that some characteristics, such as dementia diagnosis and severity, depression, anxiety, behavioral and psychological symptoms (BPSD), somatic disorders and dependency, are associated with greater unmet needs (Field et al., 2005; Hancock et al., 2006; Miranda-Castillo et al., 2010a,b).

In this context, unmet needs can be acknowledged as better predictors of the worst prognostic outcomes than the usual measures of functional or cognitive decline (Gaugler et al., 2005). These needs are also amenable to interventions that can improve health status, survival and function if followed up with active management (Stuck et al., 1993, 2004; Iliffe et al., 2004), as well as improving compliance with treatment, and quality of life (Miranda-Castillo et al., 2013). Their accurate assessment is becoming a pivotal component of effective care delivery (Challis et al., 2004) and their management a fundamental part of good health care (Ashaye et al., 2003). In order to assess and measure needs, some tools can be used for a comprehensive assessment and identification of common, important and treatable unmet needs (Iliffe et al., 2004). Once identified, the mapping of needs can be used to achieve individualized, person-centered, good-quality and effective care.

Taking this into account, the aims of the present study were to identify and describe the needs presented by an institutionalized sample and to investigate the impact of those needs on health and global functioning by analyzing their relation with other demographic and clinical characteristics.

## Materials and Methods

### Study Design and Participants

A cross-sectional multi-center study was conducted in three nursing homes in northern Portugal, between September 2012 and April 2013. A list of residents was obtained for each nursing home that agreed to participate, and all residents were considered eligible. Inclusion criteria were being a permanent resident and being able to give informed consent or assent, depending on the level of cognitive abilities. Terminally ill residents, those with delirium, who were unresponsive or unwilling to complete the assessment were excluded. For each participant a staff member was also interviewed. The participating staff member had to know the resident’s needs in order to be included.

### Data Collection and Assessments

A structured interview to collect general information on socio-demographic status, medical history and pharmacological treatment was carried out. Regarding medication, drugs were coded both as continuous and dichotomous variables (present/absent) for major categories. Anatomical Therapeutic Chemical (ATC) classification (World Health Organization, 2013) was used to indicate the anatomical main group of each drug. Comorbidities were also coded following the individual body systems of the Cumulative Illness Rating Scale for Geriatrics (CIRS-G; Miller et al., 1992).

Needs were assessed with the Camberwell Assessment of Need for the Elderly (CANE; Reynolds et al., 2000; Orrell and Hancock, 2004; range: 0–24), a comprehensive assessment tool for older people that covers 24 areas of social, physical, psychological, and environmental needs. It includes the views of the elderly, their carers and health professionals, allowing a comparison of perspectives. Overall, ratings were made by the evaluator based on the gathered information regarding the different perspectives, and each area was rated as: no need (absence of problem), met need (problem area receiving appropriate assessment or intervention), or unmet need (problem area requiring further assessment, neither receiving appropriate intervention nor receiving intervention at all). CANE presents very good validity and reliability and these properties have already been studied for the Portuguese population (Fernandes et al., 2009). Once the ratings from the evaluator were obtained for all residents (*n* = 175), only these are reported.

Study protocol also included the Mini-Mental State Examination (MMSE; Folstein et al., 1975) used as a brief measure of cognitive function (range: 0–30), Geriatric Depression Scale 15 items (GDS-15; Yesavage et al., 1983) a brief screening scale of depressive symptoms (range: 0–15), Adults and Older Adults Functional Assessment Inventory (IAFAI; Sousa et al., 2014) a Portuguese measure that assesses functional incapacity (range: 0–100%), and the European Portuguese version of Neuropsychiatric Inventory (NPI; Cummings et al., 1994) used to assess BPSD (range: 0–144). The validity and reliability of the Portuguese versions of MMSE, GDS and NPI have already been established (Guerreiro et al., 1994; Ferreira et al., 2015; Simões et al., 2015, respectively).

All protocol measures were administered in accordance with written instructions and manuals. The interviews took place in a quiet room, and separate interviews were conducted with staff members to assess the residents’ current met and unmet needs and BPSD.

For analysis proposes the cognitive decline and dementia severity was staged according MMSE ranges as: absent (MMSE of 30), questionable (26–29), mild (21–25), moderate (scores between 11 and 20) and severe dementia (MMSE score ≤10; Perneczky et al., 2006). Likewise, considering previous studies, a score ≥4 on NPI was considered indicative of clinically relevant BPSD (Lyketsos et al., 2002), and a GDS-15 score >5 was used to signal significant depressive symptoms (Simões et al., 2015).

### Ethical Considerations

The study protocol was approved by the scientific committee of the PhD Program in Clinical and Health Services Research/University of Porto. The project was approved by the three nursing home review boards (Nursing Home of Segurança Social of Porto, Instituição Particular de Solidariedade Social of Porto and Instituição Particular de Solidariedade Social of Matosinhos).

All the participants gave their written informed consent before the beginning of the assessment.

### Data Analysis

Data analyses were performed using the Statistical Package for Social Sciences (SPSS) version 20.0 for Windows. Descriptive statistics regarding demographics were calculated. Categorical variables were described through absolute frequencies, and continuous variables through mean and standard deviation (SD), median, minimum and maximum (range). Hypotheses on the distribution of continuous variables without normal distribution were tested by the non-parametric tests Mann-Whitney and Kruskal-Wallis, and to assess the strength and direction of associations between continuous variables Spearman’s correlation coefficients were calculated. Where needs were not normally distributed, non-parametric tests were chosen. All significance tests were performed at a two-tailed alpha level of 0.05.

## Results

### Sample Characteristics

The sample included 248 residents out from the eligible ones. From these, 73 residents (29.4%) were not included due to incapacity related to advanced dementia, acute illness or aphasia (*n* = 62), refusal (*n* = 1), hospitalization (*n* = 5) or death (*n* = 5) throughout the study time. The non-participants had lived on average in the homes for longer (11 vs. 7 years, *p* = 0.005) and were more severely impaired than the residents who participated. There were no significant differences regarding the average age of both groups (82 vs. 81 years, *p* = 0.361).

The mean age of the final sample (*n* = 175) was 81 (SD = 10, range: 47–103) years. Participants were mostly females (90%), widowed (51%), with a low education level (86.1% ranging from 0 to 4 years) and low socioeconomic status (77.6% low and very low status according to Graffar classification). The average length of institutionalization was 7 (SD = 11, range: 0–61) years. The main demographic and clinical characteristics of the sample are presented in Table 1.

**Table 1:** **Demographic and clinical characteristics of residents**.

Patients’ characteristics
Age, years (SD)	81 (10)
Gender, n (%)	
Male	18 (10)
Female	157 (90)
Marital status, n (%)	
Single	55 (31)
Married	12 (7)
Separated/Divorced	19 (11)
Widowed	89 (51)
Socio-economic classification (Graffar)^1^, n (%)	
Very high	1 (0.6)
High	10 (5.9)
Median	27 (15.9)
Low	68 (40.0)
Very low	64 (37.6)
Education^2^, years (SD)	3 (4)
Duration of institutionalization, years (SD)	7 (11)
Number of medications^3^, mean (SD)	7 (3)
Number of comorbidities^3^, mean (SD)	9 (4)
Cognitive impairment (MMSE)^3^, mean (SD)	22 (6)
Depression (GDS)^4^, mean (SD)	5 (4)
Functional status (IAFAI)^5^, mean (SD)	43.5 (23.5)
Behavioral and psychological symptoms (NPI)^4^, mean (SD)	6 (12)

*MMSE, Mini-Mental State Examination; GDS, Geriatric Depression Scale; IAFAI, Adults and Older Adults Functional Assessment Inventory; NPI, Neuropsychiatric Inventory. ^1^Variable with 5 missing cases; ^2^Variable with 8 missing cases; ^3^Variables with 3 missing cases; ^4^Variables with 9 missing cases; ^5^Variable with 24 missing cases*.

Most residents presented health problems with an average of 9 (SD = 4, range: 2–22) co-morbid medical conditions, and consumed medications for various purposes with a mean of 7 (SD = 3, range: 0–15). Of this sample, 86.0% consumed medication for the cardiovascular system, 79.1% for the nervous system and 68.6% for blood and blood-forming organs (Table 2).

**Table 2:** **Frequencies of consumed drugs according to ATC classification**.

ATC categories (*n* = 172)	*n* (%)
Alimentary tract and metabolism	101 (58.7)
Blood and blood forming organs	118 (68.6)
Cardiovascular system	148 (86.0)
Dermatologicals	0 (0)
Genito urinary system and sex hormones	14 (8.1)
Systemic hormonal preparations, excl. sex hormones and insulins	9 (5.2)
Antiinfectives for systemic use	3 (1.7)
Antineoplastic and immunomodulating agents	0 (0)
Musculo-skeletal system	38 (22.1)
Nervous system	136 (79.1)
Antiparasitic products, insecticides and repellents	0 (0)
Respiratory system	20 (11.6)
Sensory organs	10 (5.8)
Various	1 (0.6)

### Met and Unmet Needs

Twelve residents (6.9%) were unable to understand the CANE questions. For them, only the health professional and evaluator perspectives were obtained. Additional comparisons were conducted among those who could and could not complete CANE. Residents who were unable to complete the questionnaire were significantly more cognitively impaired (MMSE mean 22 vs. 18, *p* = 0.022). There were no significant differences regarding age (*p* = 0.159), average length of institutionalization (*p* = 0.094), depressive symptomatology (*p* = 0.065) or behavior (*p* = 0.635).

Overall 2162 needs were identified, 1523 (70.4%) were met and 639 (29.6%) unmet. The average number of needs identified was 12 (SD = 4, range: 3–18), 9 (SD = 3, range: 1–15) being met and 4 (SD = 2, range: 0–11) unmet. One hundred and seventy (97.1%) out of 175 residents presented one or more unmet needs, and the number of unmet needs did not significantly differ across the three nursing homes (mean 4 vs. 3 vs. 4, *p* = 0.514). The frequencies of CANE identified needs (met and unmet) are presented in Table 3. All residents were receiving adequate assistance for accommodation (100%), and almost all required and were receiving suitable assistance with household skills (96.6%), food (93.1%), physical health (93.1%), drugs (77.7%) and money (76.6%). The most frequent unmet needs were daytime activities (73.1%), followed by eyesight and hearing (67.4%), psychological distress (52.0%), company (40.6%) and memory (37.1%). Unmet needs presented a significant positive correlation with the age of residents (*r*_s_ = 0.236, *p* = 0.002), and a negative one with the time of institutionalization (*r*_s_ = −0.248, *p* = 0.001), but none with gender (*p* = 0.768).

**Table 3:** **Frequency of CANE needs**.

	Needs identified *n* (%)		
Need/Domains	No need	Met need	Unmet need
Accommodation	175 (100.0)	–	–
Household skills	6 (3.4)	169 (96.6)	–
Food	12 (6.9)	163 (93.1)	–
Self-care	48 (27.4)	127 (72.6)	–
Caring for other	175 (100.0)	–	–
Daytime activities	20 (11.4)	27 (15.4)	128 (73.1)
Memory	85 (48.6)	25 (14.3)	65 (37.1)
Eyesight/Hearing	31 (17.7)	26 (14.9)	118 (67.4)
Mobility	66 (37.7)	85 (48.6)	24 (13.7)
Continence	113 (64.6)	61 (34.9)	1 (0.6)
Physical health	–	163 (93.1)	12 (6.9)
Drugs	34 (19.4)	136 (77.7)	5 (2.9)
Psychotic symptoms	114 (65.1)	40 (22.9)	21 (12.0)
Psychological distress	34 (19.4)	50 (28.6)	91 (52.0)
Information	80 (45.7)	88 (50.3)	7 (4.0)
Safety (deliberate self-harm)	152 (86.9)	11 (6.3)	12 (6.9)
Safety (accidental self-harm)	66 (37.7)	102 (58.3)	7 (4.0)
Abuse/neglect	156 (89.1)	17 (9.7)	2 (1.1)
Behavior	99 (56.6)	67 (38.3)	9 (5.1)
Alcohol	168 (96.0)	4 (2.3)	3 (1.7)
Company	93 (53.1)	11 (6.3)	71 (40.6)
Intimate relationships	115 (65.7)	2 (1.1)	58 (33.1)
Money	37 (21.1)	134 (76.6)	4 (2.3)
Benefits	159 (90.9)	15 (8.6)	1 (0.6)

### Needs and Cognitive Decline

**Figure 1:**
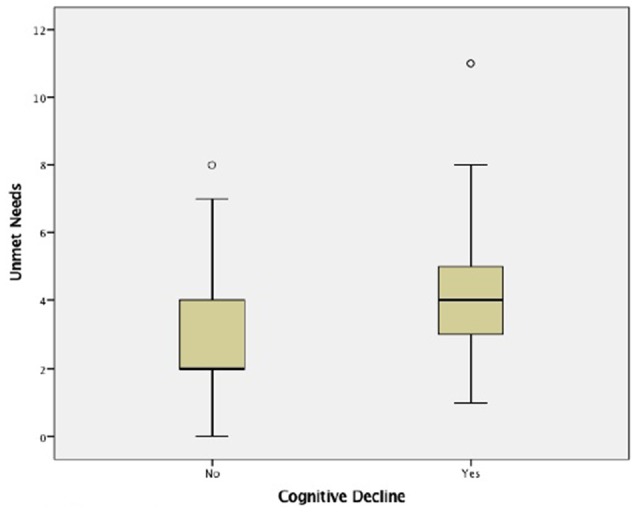
**Unmet needs considering cognitive decline**.

A total of 172 residents (98.3%) completed the MMSE. The sample scored an average of 22 (SD = 6, range: 5–30) on MMSE, and 101 (58.7%) participants scored for cognitive decline. For those with cognitive decline, the mean number of unmet needs identified was 4 (SD = 2, range: 1–11) compared to a mean of 3 (SD = 2, range: 0–8) for those without (*p* < 0.001; Figure 1). Cognitively impaired participants had unmet needs in the areas of daytime activities (*p* < 0.001), memory (*p* < 0.001) and psychotic symptoms (*p* = 0.005), that differed significantly from those without decline (Table 4). Data were also analyzed in order to compare unmet needs by severity stages of dementia as defined by Perneczky et al. (2006). Accordingly, the sample was divided into five groups: no decline (*n* = 6), questionable (*n* = 55), mild (*n* = 45), moderate (*n* = 61) and severe (*n* = 5) dementia. Table 5 presents the frequency of unmet needs identified across the stages of the disease. Early stages had more unmet needs in daytime activities (73.3%), eyesight/hearing (71.1%) and psychological distress (55.6%) domains, whereas residents in the moderate stage presented more needs related to memory (63.9%), psychotic symptoms (23.0%) and behavior (6.6%), compared to the former stage. Among the severe stage, daytime activities (100%), memory (100%) and eyesight/hearing (80.0%) were the most frequently rated unmet needs. A significant negative correlation was found between MMSE score (greater impairment) and the number of met (*r*_s_ = −0.425, *p* < 0.001), unmet (*r*_s_ = −0.369, *p* < 0.001) and global needs (*r*_s_ = −0.565, *p* < 0.001).

**Table 4:** **Unmet needs considering cognitive decline**.

	Needs identified *n* (%) Cognitive decline	
Need/Domains	Without (*n* = 71)	With (*n* = 101)	*p*-value^1^
Accommodation	−	−	−
Household skills	−	−	−
Food	−	−	−
Self-care	−	−	−
Caring for other	−	−	−
Daytime activities	40 (56.3)	85 (84.2)	<0.001
Memory	5 (7.0)	57 (56.4)	<0.001
Eyesight/Hearing	48 (67.6)	67 (66.3)	0.161
Mobility	6 (8.5)	17 (16.8)	0.267
Continence	0 (0)	1 (1.0)	<0.001
Physical health	4 (5.6)	7 (6.9)	0.766
Drugs	2 (2.8)	3 (3.0)	<0.001
Psychotic symptoms	3 (4.2)	17 (16.8)	0.005
Psychological distress	37 (52.1)	51 (50.5)	0.061
Information	0 (0)	4 (4.0)	<0.001
Safety (deliberate self-harm)	6 (8.5)	5 (5.0)	0.777
Safety (accidental self-harm)	2 (2.8)	5 (5.0)	0.001
Abuse/neglect	1 (1.4)	1 (1.0)	0.901
Behavior	1 (1.4)	6 (5.9)	0.254
Alcohol	2 (2.8)	1 (1.0)	0.596
Company	26 (36.6)	43 (42.6)	0.393
Intimate relationships	22 (31.0)	35 (34.7)	0.874
Money	0 (0)	4 (4.0)	<0.001
Benefits	1 (1.4)	0 (0)	0.259

*^1^Chi-Square test*.

**Table 5:** **Unmet needs across severity stages of dementia**.

		Needs identified *n* (%)	
Need/Domains	No (*n* = 6)	Questionable (*n* = 55)	Mild (*n* = 45)	Moderate (*n* = 61)	Severe (*n* = 5)
Accommodation	−	−	−	−	−
Household skills	−	−	−	−	−
Food	−	−	−	−	−
Self-care	−	−	−	−	−
Caring for other	−	−	−	−	−
Daytime activities	3 (50.0)	31 (56.4)	33 (73.3)	53 (86.9)	5 (100.0)
Memory	0 (0)	1 (1.8)	17 (37.8)	39 (63.9)	5 (100.0)
Eyesight/Hearing	1 (16.7)	39 (70.9)	32 (71.1)	39 (63.9)	4 (80.0)
Mobility	1 (16.7)	3 (5.5)	5 (11.1)	11 (18)	3 (60.0)
Continence	0 (0)	0 (0)	0 (0)	1 (1.6)	0 (0)
Physical health	0 (0)	1 (1.8)	5 (11.1)	5 (8.2)	0 (0)
Drugs	0 (0)	2 (3.6)	1 (2.2)	2 (3.3)	0 (0)
Psychotic symptoms	0 (0)	4 (7.3)	1 (2.2)	14 (23)	1 (20.0)
Psychological distress	3 (50)	27 (49.1)	25 (55.6)	31 (50.8)	2 (40.0)
Information	0 (0)	0 (0)	0 (0)	3 (4.9)	1 (20.0)
Safety (deliberate self-harm)	1 (16.7)	3 (5.5)	5 (11.1)	2 (3.3)	0 (0)
Safety (accidental self-harm)	0 (0)	3 (5.5)	1 (2.2)	3 (4.9)	0 (0)
Abuse/neglect	1 (16.7)	0 (0)	0 (0)	1 (1.6)	0 (0)
Behavior	0 (0)	1 (1.8)	1 (2.2)	4 (6.6)	1 (20.0)
Alcohol	0 (0)	3 (5.5)	0 (0)	0 (0)	0 (0)
Company	1 (16.7)	21 (38.2)	15 (33.3)	31 (50.8)	1 (20.0)
Intimate relationships	2 (33.3)	17 (30.9)	13 (28.9)	24 (39.3)	1 (20.0)
Money	0 (0)	0 (0)	2 (4.4)	2 (3.3)	0 (0)
Benefits	0 (0)	1 (1.8)	0 (0)	0 (0)	0 (0)

### Needs and Depressive Symptoms

**Figure 2:**
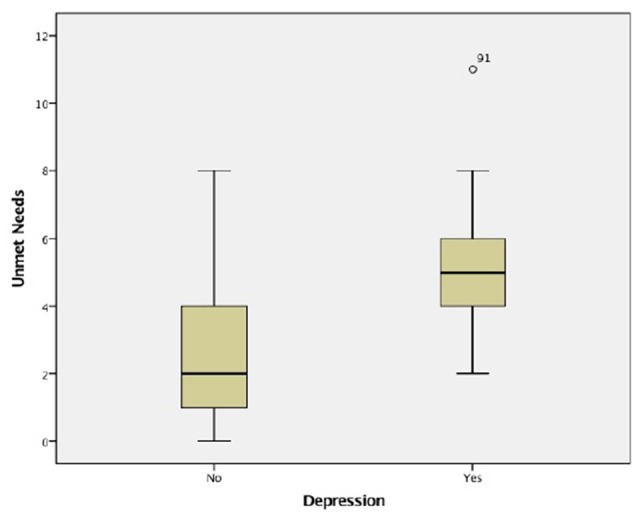
**Unmet needs considering depressive symptoms**.

Of the 175 participants, 166 (94.9%) completed the GDS-15, and their average score was 5 (SD = 6, range: 0–14). The study sample was also divided on the basis of GDS score. From the whole sample, 75 residents (45.2%) scored for depression (GDS scores ranged from 5 to 15). Unmet needs were more common in residents who scored for depression. Elderly with depression presented a mean of 5 unmet needs (SD = 2, range: 2–11) compared to those without who had a mean of 3 (SD = 2, range: 0–8; *p* < 0.001; Figure 2). Considering the presence of depressive symptoms, psychological distress (*p* < 0.001), daytime activities (*p* = 0.022), company (*p* < 0.001) and intimate relationships (*p* < 0.001) domains differed significantly between the groups (Table 6). Data also showed significant correlations between the presence of depressive symptoms assessed by GDS and unmet (*r*_s_ = 0.683, *p* < 0.001) and global needs (*r*_s_ = 0.407, *p* < 0.001), while no significant association were found with met needs (*r*_s_ = 0.011, *p* = 0.889).

**Table 6:** **Unmet needs considering depressive symptoms**.

	Needs identified *n* (%)	
Need/Domains	No depression (*n* = 91)	Depression (*n* = 75)	*p*-value^1^	
Accommodation	−	−	−
Household skills	−	−	−
Food	−	−	−
Self-care	−	−	−
Caring for other	−	−	−
Daytime activities	58 (63.7)	61 (81.3)	0.022
Memory	32 (35.2)	27 (36.0)	0.72
Eyesight/Hearing	55 (60.4)	55 (73.3)	0.172
Mobility	11 (12.1)	12 (16.0)	0.078
Continence	0 (0)	1 (1.3)	0.326
Physical health	3 (3.3)	7 (9.3)	0.188
Drugs	1 (1.1)	4 (5.3)	0.166
Psychotic symptoms	6 (6.6)	14 (18.7)	0.013
Psychological distress	19 (20.9)	68 (90.7)	<0.001
Information	2 (2.2)	2 (2.7)	0.953
Safety (deliberate self-harm)	1 (1.1)	10 (13.3)	0.002
Safety (accidental self-harm)	2 (2.2)	5 (6.7)	0.322
Abuse/neglect	1 (1.1)	1 (1.3)	0.276
Behavior	4 (4.4)	3 (4.0)	>0.999
Alcohol	2 (2.2)	1 (1.3)	>0.999
Company	14 (15.4)	53 (70.7)	<0.001
Intimate relationships	17 (18.7)	39 (52.0)	<0.001
Money	2 (2.2)	2 (2.7)	0.842
Benefits	0 (0)	1 (1.3)	0.211

*^1^Chi-Square test*.

### Needs and Behavioral and Psychological Symptoms

**Figure 3:**
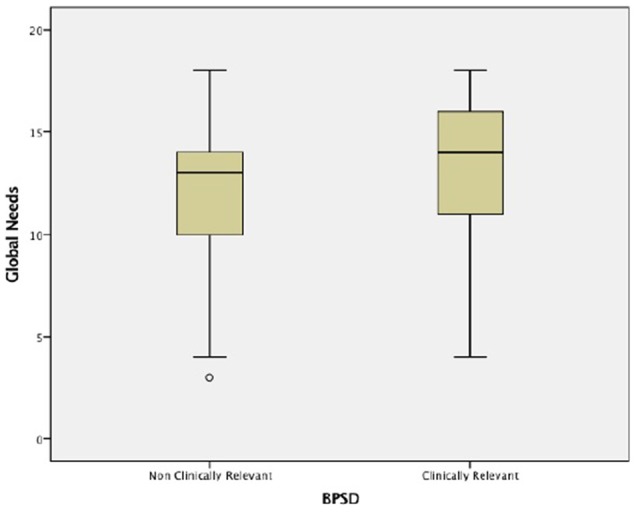
**Global needs considering behavioral and psychological symptoms**.

Concerning BPSD, at screening 50.6% of the sample presented at least one symptom, and 56 (33.7%) scored above the NPI cut-off for clinical significance. The average NPI score was 6 (SD = 12, range: 0–76). The most common BPSD across the sample were sleep and nighttime behavior change (54%), delusions (22%), dysphoria/depression (19%), irritability/lability (17%) and agitation/aggression (15%), while the least prevalent were elation/euphoria (3%), aberrant motor behavior (4%) and disinhibition (5%). These results have been described in detail elsewhere (Ferreira et al., 2015). Those with more BPSD had significantly more global needs (mean 14 vs. 13, *p* = 0.008; Figure 3), particularly in the areas of psychotic symptoms (*p* < 0.001), behavior (*p* < 0.001) and mobility (*p* = 0.006; Table 7). Significant correlations between the presence of BPSD and unmet and global needs (*r*_s_ = 0.181, *p* = 0.020; *r*_s_ = 0.254, *p* = 0.001, respectively) were found, but not with those being met (*r*_s_ = 0.152, *p* = 0.051).

**Table 7:** **Unmet needs considering behavioral and psychological symptoms**.

	Needs identified *n* (%)	
Need/Domains	Non clinically relev. (*n* = 110)	Clinically relev. (*n* = 56)	*p*-value^1^	
Accommodation	−	−	−
Household skills	−	−	−
Food	−	−	−
Self-care	−	−	−
Caring for other	−	−	−
Daytime activities	75 (68.2)	46 (82.1)	0.151
Memory	37 (33.6)	26 (46.4)	0.15
Eyesight/Hearing	74 (67.3)	38 (67.9)	0.939
Mobility	15 (13.6)	8 (14.3)	0.006
Continence	0 (0)	1 (1.8)	0.486
Physical health	10 (9.1)	2 (3.6)	0.227
Drugs	3 (2.7)	2 (3.6)	0.654
Psychotic symptoms	3 (2.7)	18 (32.1)	<0.001
Psychological distress	57 (51.8)	31 (55.4)	0.105
Information	4 (3.6)	3 (5.4)	0.237
Safety (deliberate self-harm)	7 (6.4)	5 (8.9)	0.596
Safety (accidental self-harm)	5 (4.5)	2 (3.6)	0.601
Abuse/neglect	2 (1.8)	0 (0)	0.583
Behavior	1 (0.9)	8 (14.3)	<0.001
Alcohol	1 (0.9)	2 (3.6)	0.197
Company	43 (39.1)	25 (44.6)	0.265
Intimate relationships	38 (34.5)	18 (32.1)	0.566
Money	1 (0.9)	3 (5.4)	0.19
Benefits	1 (0.9)	0 (0)	>0.999

*^1^Chi-Square test*.

### Needs and Functional Dependency

Of the 175 participants, 151 (86.3%) completed the IAFAI, and their average score was 43.5% (SD = 23.5%, range: 0–93.6%). The functional dependency was correlated with greater unmet needs. Significant correlations between functional dependency and met (*r*_s_ = 0.642, *p* < 0.001), unmet (*r*_s_ = 0.505, *p* < 0.001) and global needs (*r*_s_ = 0.796, *p* < 0.001) were found.

## Discussion

The main aim of the present study was to describe the met and unmet needs of residents in nursing homes. To our knowledge this is the first study systematically conducted with this purpose in the northern Portugal.

Needs assessment has become a central issue following the growing recognition that it could lead to more appropriate and effective provision of care, services and resource usage (Worden et al., 2006). Overall, and in line with previous studies (Holmquist et al., 2003; Hancock et al., 2006), unmet needs were prevalent across these three nursing homes, and clustered in particular domains, namely the psychosocial, such as daytime activities, company and psychological distress (Martin et al., 2002; Hancock et al., 2006; Cohen-Mansfield et al., 2015). Needs rated in the areas of daytime activities and company, are a trend in these long-term care settings that has been encountered by previous researchers (Mozley et al., 2000; Hancock et al., 2006; Orrell et al., 2008; Popham and Orrell, 2012) and systematic reviews (e.g., Cadieux et al., 2013). Sensory needs and memory were also found to be among the most prevalent in this sample, in accordance with findings by Hancock et al. (2006). Memory was also the highest ranked unmet domain (80.2%) found by Iliffe et al. (2004) in their sample of elderly residents. The present results are also in accordance with previous Portuguese studies conducted in psychiatry and mental health services in northern Portugal (Fernandes et al., 2009; Passos et al., 2012) regarding the most common unmet domains, except for eyesight/hearing found in the present study, and for social benefits and continence areas found by Passos et al. (2012).

A negative relationship between lengths of residency and the number of unmet needs was also found, which indicates that the longer the resident had lived in the home, the less their unmet needs were rated. Environmental and physical health needs were generally met, as had already been found by others not only in long-term care (Martin et al., 2002; Hancock et al., 2006; Orrell et al., 2008) but also in other settings like primary care (e.g., Walters et al., 2000). Those needs of a more basic or instrumental nature, such as accommodation, self-care, or food preparation, seem consistently met, apart from any specific psychopathology or disability, suggesting that long-term care settings are effective in identifying and meeting them.

The number of unmet needs presented by this sample is greater than those reported by other studies conducted in long term care (Martin et al., 2002; van der Ploeg et al., 2013; Cohen-Mansfield et al., 2015) and in other settings such as the community (van der Roest et al., 2009; Miranda-Castillo et al., 2010a,b), primary care (Hoogendijk et al., 2014), sheltered accommodation (Field et al., 2005) or psychiatric day hospitals (Ashaye et al., 2003). However, they agree with the data found by Orrell et al. (2007) at baseline in both their experimental and control groups (4.77 and 4.12 unmet needs on average, respectively), by Hancock et al. (2006; 4.4 unmet needs on average per resident), or by Greaves et al. (2006; 4.46 unmet needs found in a sample of patients that were referred to a psychiatric liaison service). Differences between settings agree with the fact that in institutional care, residents present a higher prevalence of other concurrent problems, such as depression, behavior, physical dependency and social problems (Fahy and Livingston, 2001). On the other hand, differences within long term-care settings may reflect different inclusion and referral criteria, as well as variance in the applied study designs and methodologies.

As noted by Miranda-Castillo et al. (2013), these consistent findings from different studies and settings should be a matter of concern. In fact, there are specific areas or domains in which unmet needs are more likely to occur, namely the domains of psychological distress, company and daytime activities, which are consistently reported as unmet (de Boer et al., 2007; van der Roest et al., 2007, 2009; Orrell et al., 2008; Miranda-Castillo et al., 2010a,b; von Kutzleben et al., 2012; Hoogendijk et al., 2014; Mazurek et al., 2015), and yet they tend to remain unmet despite this. Bearing this in mind, these findings convey an important message concerning the importance of effective management of mood disorders in nursing care, along with the involvement of the elderly in their own care (Orrell et al., 2008). It is worth noting that even residents who scored for moderate or severe decline in MMSE also gave pertinent information on their needs, and those also converge mainly in psychosocial areas, which has already been confirmed by other researchers (e.g., Orrell et al., 2008; Popham and Orrell, 2012). In fact, most residents are able to understand what people say or to interact sociably (Rocha et al., 2013), suggesting that the encouragement of multi-dimensional activities and participation could help to address some of those residents’ unmet needs.

The study also sought to determine whether the number of needs was related to other important variables. Overall, in this sample more unmet needs were associated with the worst outcomes measured. In line with other studies, the presence of unmet needs was associated with increased cognitive and functional decline (Martin et al., 2002), as well as with more depressive symptoms (Field et al., 2005; Hancock et al., 2006; Mazurek et al., 2015) and BPSD (Hancock et al., 2006; Miranda-Castillo et al., 2010a). Unmet needs were positively associated with the number of BPSD, and it is worth noting that these symptoms can predict cognitive and functional decline, higher unmet needs (Miranda-Castillo et al., 2010a,b), are associated with the current and future disease progression (Wadsworth et al., 2012), as well as tending to boost premature institutionalization (Yaffe et al., 2002; Herrmann et al., 2006; Scarmeas et al., 2007). In this sample, a high proportion of residents 80.8% presented psychiatric morbidity, which also comprises depressive symptoms. The high level of depressive symptomatology found (45.2%) is consistent with other cross-sectional studies (e.g., Mozley et al., 2000). Depression has not only been described as the commonest psychiatric disorder of later life (Katz et al., 1989), which can exist in association with other significant medical conditions, but has also been shown to be more prevalent in nursing home residents. It is important to verify that depression in nursing homes is heterogeneously presented and some of its symptoms such as apathy, withdrawal or disengagement are also considered as nonspecific signs of deterioration (Katz et al., 1989).

It is noteworthy that except for the age of residents, unmet needs were positively correlated with modifiable or treatable characteristics such as the presence of depressive symptoms or behavioral problems. These are areas that have also been found amenable to interventions planned by mental health professionals (Orrell et al., 2007), rather than relying solely on pharmacological approaches (Fossey et al., 2006). Therefore, appropriate assessment and interventions focused on these factors could provide opportunities to decrease the frequency of unmet needs (Miranda-Castillo et al., 2010a; Cadieux et al., 2013).

The study has some potential limitations and caution should be exercised upon generalization of the present findings. Firstly, its cross-sectional design, with findings that may point toward some important relations but that cannot imply causality. Considering the inclusion and exclusion criteria that were fixed, it is possible that those elderly with more unmet needs were under-recruited due to their incapacity to respond to the evaluation. In this way, the present non-random sample may be less disabled than the actual institutionalized population, which may have led to an underestimation of the real number of needs. Since the residents’ participation was voluntary, it may have induced a positive bias into the findings. Finally, despite the discrepancy of the gender ratio presented in this sample, it is in line with other studies conducted in comparable settings (e.g., Hancock et al., 2006).

Despite the referred limitations, the study also has strengths. Firstly, valid and reliable measures were used. Secondly, the participants were included from various stages of dementia decline. This continuum is thought to represent and capture the range of needs over time in the disease progression. Thirdly, the study included a relatively large sample of residents and a detailed and standardized tool to access needs was applied. Considering the prognostic value of unmet needs, their evaluation should become a standard part of clinical evaluation providing important information to professionals, elderly and caregivers. Finally, as already noted in other studies (Walters et al., 2000; Orrell et al., 2007) CANE was also used as a means for coaching care staff about the range of needs and potential interventions.

Most studies have assessed needs using a cross-sectional approach (e.g., Field et al., 2005; Hancock et al., 2006; van der Ploeg et al., 2013), thus the longitudinal role of unmet needs is less well defined. Considering that the elderly population has been steadily increasing, further studies should not only include more homogeneous care facilities and larger samples, but also should imply prospective longitudinal approaches and focus on the relationships between unmet needs and clinical variables.

In conclusion, the present study was a contribution to the characterization of needs in nursing homes, which should be promoted in order to improve strategies for future care with different and complementary perspectives integrated into collaborative and tailored elderly care plans.

## Author Contributions

LF defined and designed the study and supervised the data collection. ARF collected the data. LF and ARF drafted the article. CCD carried out the statistical analyses. All the authors contributed to the interpretation of the data, revision of the article, and approved the final manuscript.

## Funding

Novartis Farma sponsored the data collection and statistical analysis. The publication was supported by FEDER through Programa Operacional Competitividade e Internacionalização – COMPETE2020 and by National Funds through FCT – Fundação para a Ciência e a Tecnologia within CINTESIS, R&D Unit (reference UID/IC/4255/2013). The sponsors did not play any role in the design, methods, data collection and analyses, or in the preparation of the article.

## Conflict of Interest Statement

The authors declare that the research was conducted in the absence of any commercial or financial relationships that could be construed as a potential conflict of interest.
